# RETRACTED: Nazir et al. Spirulina Unleashed: A Pancreatic Symphony to Restore Glycemic Balance and Improve Hyperlipidemia and Antioxidant Properties by Transcriptional Modulation of Genes in a Rat Model. *Foods* 2024, *13*, 3512

**DOI:** 10.3390/foods14193368

**Published:** 2025-09-29

**Authors:** Anum Nazir, Mahr un Nisa, Muhammad Abdul Rahim, Isam A. Mohamed Ahmed, Moneera O. Aljobair

**Affiliations:** 1 Department of Nutritional Sciences, Faculty of Medical Sciences, Government College University, Faisalabad 38000, Pakistan; marzixanu@gmail.com; 2 Department of Food Science & Nutrition, Faculty of Medicine and Allied Health Sciences, Times Institute, Multan 60700, Pakistan; 3 Department of Food Science and Nutrition, College of Food and Agricultural Sciences, King Saud University, Riyadh 11451, Saudi Arabia; 4 Department of Physical Sport Sciences, College of Sports Sciences & Physical Activity, Princess Nourah Bint Abdulrahman University, Riyadh 11671, Saudi Arabia; moaljobair@pnu.edu.sa

The journal retracts the article “Spirulina Unleashed: A Pancreatic Symphony to Restore Glycemic Balance and Improve Hyperlipidemia and Antioxidant Properties by Transcriptional Modulation of Genes in a Rat Model” [1] cited above.

Following publication, concerns were brought to the attention of the Editorial Office regarding partial duplications contained within images presented in this article [1].

Adhering to our standard procedure, an investigation was conducted by the Editorial Office and Editorial Board that identified indications of inappropriate editing and partial duplication between figures presented in this article [1]. Although the authors cooperated with the investigation, they were unable to provide a satisfactory explanation for the overlap or submit a raw image file for the Editorial Board to verify the integrity of the images. Consequently, the Editorial Board has lost confidence in the reliability of the findings and has decided to retract this publication, as per MDPI’s retraction policy (https://www.mdpi.com/ethics#_bookmark30, accessed on 6 August 2025).

This retraction was approved by the Editor-in-Chief of the *Foods* journal.

The authors did not agree to this retraction.
